# Molecular Intricacies and the Role of ER Stress in Diabetes

**DOI:** 10.1155/2012/958169

**Published:** 2012-06-04

**Authors:** Muthuswamy Balasubramanyam, Lalit P. Singh, Sampathkumar Rangasamy

**Affiliations:** ^1^Department of Cell & Molecular Biology, Madras Diabetes Research Foundation (MDRF), 4 Conran Smith Road, Gopalapuram, Chennai 600086, India; ^2^Department of Anatomy and Cell Biology and Department of Ophthalmology, Wayne State University School of Medicine, Detroit, MI 48201, USA; ^3^Department of Cell Biology and Physiology, University of New Mexico School of Medicine, Albuquerque, NM 87131, USA

Diabetes mellitus is a metabolic disease caused by both genetic and environmental factors. The pathogenic mechanism(s) of diabetes are complex, and the complicated networks related to this disease involve distinct signaling pathways. Evidence has recently been provided that ER stress might be involved in the pathogenesis of diabetes and its complications. Early steps in the maturation of secretory proteins take place in the ER, for example, the folding of the nascent polypeptide chains and posttranslational modifications important for proper folding and function of the protein. At a stage (due to several metabolic disturbances), when unfolded polypeptide exceeds the folding and/or processing capacity of the ER, cells are susceptible to a phenomenon referred to as “ER stress.” Under these conditions, specific signaling pathways, termed the unfolded protein response (UPR), are activated to return the ER to its normal physiological state. Prolonged activation of the ER stress and the UPR can lead to cell pathology and subsequent tissue dysfunction. There is now ample evidence that the UPR is chronically activated in many disease states including diabetes and its complications. Therefore, a better understanding of the pathways regulating ER stress and UPR is warranted in order to be instrumental for the design of novel therapies for diabetes and its complications.

In this focused issue of the journal, we have assembled several invited reviews, from well-recognized experts in their fields, as well as original research articles. These reviews provide state-of-the-art knowledge dealing with several mechanisms not only related to the genesis of diabetes but also to its progression to diabetic complications, all of which potentially originate or converge from chronic ER stress. In addition, several excellent original research articles demonstrate novel pathophysiologic aspects of diabetes with mechanistic studies central to ER stress and give hope and directionality for identifying new drug targets and developing newer therapeutic measures.

Of all the professional secretory cells we possess, *β*-cells are the most sensitive to ER stress because of the large fluctuations in protein synthesis (including insulin) they face daily. M.-K. Kim et al. have reviewed how this “protein quality-control machinery” of the cell is responsible for appropriate insulin biosynthesis and how ER stress plays an important role in the impairment of insulin biosynthesis. J. Zhong et al. have summarized the status on how ER stress plays an essential role in autoimmune-mediated *β*-cell destruction and also pointed out how ER stress regulates the functionality of immune cells relevant to autoimmune progression during Type 1 diabetes development. In an attempt to improve islet transplantation in humans, the molecular mechanism of apoptosis in *β* cells of islets in the transplantation setting needs to be clearly understood. In this context, M. Wang et al. have discussed their original research work on human islets subjected to multiple stressors and delineated several apoptotic pathways originating from oxidative stress, autophagy, and ER stress.

While ER stress is emerging as a unifying paradigm in diabetes and its complications, several recent studies, emphasized a definite role of ER stress in retinal, podocyte, and neuronal cell apoptosis. G. Jing et al. have summarized the recent progress on ER stress and apoptosis in retinal diseases, focusing on various proapoptotic and antiapoptotic pathways that are activated by the UPR and discussed how these pathways contribute to ER stress-induced apoptosis in retinal cells. Considering the fact that ER stress is initially an adaptive response, studying ER stress-related factors appear to unravel novel drug targets to prevent and treat diabetic retinopathy. In this connection, W. -K. Hu et al. have explained the role of P58IPK and ER-associated degradation (ERAD) of unfolded protein which prevents ER stress and reduce retinal vascular leakage under high-glucose conditions. While thioredoxin interacting protein (TXNIP) has been recently identified as an early response gene highly induced by diabetes and hyperglycemia, its role in the pathogenesis of diabetic retinopathy is not clearly understood. Using appropriate animal model and retinal Muller cell line and several molecular biology techniques, T. S. Devi et al. have described how upregulation of TXNIP evokes a program of cellular defense and survival mechanism(s) that ultimately lead to oxidative stress, ER-stress, inflammation, and apoptosis.

Despite a great deal of research, the mechanisms that may link high-glucose concentrations to the molecular and cellular pathways of diabetic atherogenesis are not fully understood. D. R. Beriault and G. H. Werstuck have summarized the current state of our knowledge of pathways and mechanisms that may link diabetes and hyperglycemia to atherogenesis highlighting the recent work from their lab (and others) that supports a role for ER stress in these processes. Although recent studies have shown that perturbations in lipid metabolism cause an ER stress response, very little is known about the mechanism of UPR activation by perturbations in glucose and lipid metabolism. Moreover, it has been demonstrated that 4-phenylbutyrate (4-PBA) and tauroursodeoxycholic acid (TUDCA), which are two different chemical structures having chemical chaperone activity in common, relieve ER stress. Using THP-1 human monocytes as a surrogate cell model and utilizing several molecular biology techniques, R. Lenin et al. have demonstrated that monocytes subjected to glucolipotoxicity exhibited increased UPR responses (as evidenced by increased mRNA expression of several ER stress markers) along with increased oxidative stress and apoptosis. Interestingly, ER stress inducted by glucolipotoxicity was shown resisted by PBA. These observations constitute an important proof of principle that manipulation of the ER system to decrease ER stress by chemical agents may have therapeutic implications for diabetes and its complications.

Lastly, the prevalence of nonalcoholic fatty liver disease (NAFLD) has increased in parallel with the epidemics of obesity and type 2 diabetes, which are risk factors for NAFLD. Whereas the association of type 2 diabetes with microvascular complications and macrovascular disease is well established, the association of type 2 diabetes with NAFLD is only recently recognized and so are the inter-related pathogenic mechanisms. Using steatohepatitis animal model and HepB3 cells, M. K. Chae et al. have demonstrated that Pentoxifylline (a known anti-inflammatory agent) attenuates methionine and choline-1-deficient diet-induced steatohepatitis by suppressing ER stress.

These papers, hopefully, will provide better understanding of ER stress and UPR pathway involvement in the pathogenesis of diabetes and its complications and bring forward new and innovative ideas with respect to the development of efficient and adjuvant treatment modalities. Considering the involvement of ER stress in multiple tissues and their convergence in multiple pathogenic pathways (oxidative stress, inflammation, apoptosis, autophagy, and proteasomal degradation), targeting the ER stress pathway appears as a promising therapeutic strategy. The significant side effects with existing drugs and the demand for newer molecules with improved safety and a different mode of action justifies this directionality. In fact, it has been reported that chemical ER chaperones can reduce ER stress, suggesting that small molecules can affect ER stress signaling in disease states. There is also much hope in investigating the traditional plant principles of medicinal claims to see whether they act as beneficial ER stress modulators. Given the possible development of novel UPR-targeted therapies for diabetes and its complications, it is essential to know which components of the ER stress response to target and which particular disease stage will be most amenable to therapy.

Although there is an enormous progress in studying the ER stress aspects, the list of unresolved queries in the stress mediated pathway of ER dysfunction in diabetes and its complications warrant continued research efforts. Obviously not all aspects of this exciting ER stress field could be addressed in one issue and we extend our apologies to many contributors of this field whose work has not been covered. We thank all the authors who contributed to this special issue of EDR and the reviewers for the highly constructive and helpful comments.

*Muthuswamy Balasubramanyam* *Muthuswamy Balasubramanyam* *Lalit P. Singh* *Lalit P. Singh* *Sampathkumar Rangasamy* *Sampathkumar Rangasamy*

